# Qualitative Studies on Implicit Criteria during the Individualized Selection Procedure for Medical Studies at Witten/Herdecke University (UW/H)

**DOI:** 10.3205/zma001211

**Published:** 2019-02-15

**Authors:** Michaela Zupanic, Jan P. Ehlers, Julia Fricke, Ruth-Maria Gerken, Marzellus Hofmann, Janina Nitsche, Martin R. Fischer, Daniel Bauer

**Affiliations:** 1 Witten/Herdecke University, Faculty of Health, Personality Psychology and Diagnostics, Witten, Germany; 2 Witten/Herdecke University, Faculty of Health, Student Dean Office, Witten, Germany; 3 General Practitioner Dr. Louis Bonvin, Crans Montana, Switzerland; 4 Katholische Kliniken im Märkischen Kreis, Iserlohn, Germany; 5 Medical Center Bad Endorf, Bad Endorf, Germany; 6 University Hospital, Institute for Medical Education, Munich, Germany; 7 University of Bern, Faculty of Medicine, Institute of Medical Education, Bern, Switzerland

**Keywords:** student selection for medical studies, Witten/Herdecke University, selection criteria, Master Plan Medical Studies 2020, ability to reflect

## Abstract

**Objective: **The individualized two-stage selection procedure for medical studies at Witten/Herdecke University (UW/H) has been in use for more than 30 years and comprises explicit and implicit selection criteria. This analysis aims at identifying the implicit criteria and answering the question whether an internal consistency of these implicit criteria may be verified for the different phases of the selection procedure (when evaluating the statements of purpose, during the selection weekend and during the concluding discussions of assessors).

**Methodology:** Three qualitative studies on all phases of the selection procedure at UW/H have been conducted for determining the implicit assessment criteria of assessors:

1. statements of purpose in extreme group comparison (12 admissions versus 18 rejections);
2. semi-structured expert interviews (N=25) on the selection weekend;
3. focus group analysis of the concluding discussions on two selection weekends (N=16).

**Results: **The content analysis of the statements of purpose yielded 14 main categories with significant deviations between extreme groups in the categories school career, reasons for application and reflections as well as regarding the higher education entrance qualification grade. Based on the expert interviews, three main categories could be identified: intellectual ability, motivation and social competence, and the ability to reflect as a cross-content category. The focus group analysis yielded four main categories: performance, personality, personal growth potential and ability to reflect. Most frequently, the ability to reflect was mentioned as an assessment criterion.

**Conclusion: **The main assessment categories are: motivation for the medical profession and starting studies at UW/H; performance and scholastic aptitude; personality, personal growth potential and social competence, as well as the ability to reflect as the most important basic competence and general category. Assessors consider the ability to reflect as a predictor of lifelong professional development as a physician.

## Zusammenfassung

**Zielsetzung: **Das seit über 30 Jahren etablierte, individualisierte zweistufige Auswahlverfahren Humanmedizin der Universität Witten/Herdecke (UW/H) umfasst explizite und implizite Auswahlkriterien. Zielsetzung dieser Analyse sind die Identifikation der impliziten Kriterien und die Beantwortung der Fragestellung, ob eine interne Konsistenz dieser impliziten Kriterien in den verschiedenen Phasen des Auswahlverfahrens (bei der Bewertung der Motivationsschreiben, während des Auswahlwochenendes und bei den Abschlussgesprächen der Gutachtenden) belegt werden kann.

**Methodik: **Drei qualitative Untersuchungen zu allen Phasen des Auswahlverfahrens an der UW/H wurden zur Ermittlung der impliziten Bewertungskriterien der Gutachtenden durchgeführt:

1. Motivationsschreiben im Extremgruppenvergleich (12 Zusagen versus 18 Absagen);
2. teilstrukturierte Experteninterviews (N=25) zum Auswahlwochenende;
3. Fokusgruppenanalyse zur Abschlussdiskussion an zwei Auswahlwochenenden (N=16).

**Ergebnisse: **Inhaltsanalytisch ergaben sich bei den Motivationsschreiben 14 Hauptkategorien mit deutlichen Unterschieden zwischen den Extremgruppen in den Kategorien Schullaufbahn, Bewerbungsgründe und Reflexionen sowie in der Abiturnote. Aus den Experteninterviews wurden die drei Hauptkategorien intellektuelle Fähigkeiten, Motivation und soziale Kompetenzen identifiziert sowie die Reflexionsfähigkeit als inhaltlich übergreifende Kategorie. Die Fokusgruppenanalyse ergab die vier Hauptkategorien Leistung, Persönlichkeit, Entwicklungsfähigkeit und Reflexionsfähigkeit. Die Reflexionsfähigkeit wurde dabei am häufigsten als Bewertungskriterium genannt.

**Schlussfolgerung: **Hauptkategorien der Bewertung sind die Motivation für den Arztberuf und das Studium an der UW/H; Leistung und Studierfähigkeit; Persönlichkeit, Entwicklungsfähigkeit und soziale Kompetenz sowie Reflexionsfähigkeit als wichtigste zugrunde liegende Kompetenz und übergreifende Kategorie. Die Reflexionsfähigkeit gilt dabei aus Sicht der Gutachterinnen und Gutachter als Prädiktor für eine lebenslange professionelle Entwicklung als Ärztin oder Arzt.

## Introduction

Applicants for medical studies often have idealistic motives and would like to become a good physician [1]. A clear definition of a good physician, however, does not exist. Instead, there are extensive requirements: A good physician shall be an expert in his/her field and act professionally, i.e. in an ethically correct way, honestly and empathetically, and have good communication skills for confidential doctor-patient relationships and interaction in interdisciplinary teams [2]. The selection procedure for medical studies, i.e. the path towards reaching the decision on who will be admitted, is an important and controversial issue being discussed on the international [3], [4] and the national scale, as well as from scientific [5], political [6], [7] and journalistic [8] perspectives. 

In Germany an average of five applicants apply for a place to study medicine in winter terms, while 11 applicants apply for a place to study medicine in summer terms [9]. The waiting period is up to 14 terms in some cases [10]. Selection procedures shall ensure a minimization of drop-outs [11] as well as the selection of the best students with regard to completing studies within the standard period of study and the anticipated professional competence [12]. The same quality requirements apply as to all test procedures: prognostic validity, objectivity, reliability, fairness, transparency, acceptance and practicability [13]. 

On an international scale, different procedures for selecting students are researched and used. These procedures often consult results of previous academic performance due to their satisfactory prognostic validity [14], [15]. School-leaving grades or higher education entrance qualifications being used as selection criteria, however, are criticized for not being objective and reliable, and for not substantiating an aptitude for the respective degree course [16], [17]. Selection interviews [18], scholastic aptitude tests [19] and statements of purpose [20] are also well established. 

After deduction of preset quotas, 20% of the places to study medicine in Germany are allocated according to the grade point average of the higher education entrance qualification, 20% according to the number of waiting terms and the remaining 60% via the university’s individual selection procedure (ISP). During the ISP further methods such as interviews [21] or scientific aptitude tests [22] are employed while the higher education entrance qualification grade is still primarily taken into account [10].

As a private non-profit medical education institution Witten/Herdecke University (UW/H) is in the position to conduct its own selection procedure independent of federal and state admissions regulations. The degree course corresponds to a model curriculum according to § 41 Medical Practice Regulations. At the beginning of the present studies, the two-stage selection procedure at UW/H took place as follows [23]: Admission requirements included the higher education entrance qualification and a six-month nursing internship to be completed before students started their studies. An initial selection was made on the basis of a written application (statement of purpose, tabular and detailed curriculum vitae, written exercise varying depending on the application cycle). The final decision was taken based on interviews conducted during a selection weekend. On three weekends, 48 applicants each were invited to introduce themselves. All participants took part in one individual interview each on their motivation and their curriculum vitae as well as in six group discussions. During group discussions, participants presented a topic of their own choice. In addition, their behavior in debate on the presentations of other participants was assessed.

For the last 25 years, this UW/H selection procedure has been examined over and over again for quality assurance reasons [24], [25] and subsequently further adjusted in order to better meet the ambition to admit the most suitable applicants for studying at UW/H. The factors for admission were determined through a path analysis in 2007 [24]. As a result, a higher education entrance qualification grade of 1.3 constituted an increased chance of admission. However, the grade point average of the higher education entrance qualification was with 2.0 only slightly better in case of admissions than the one of all applicants during the first written stage of the procedure (2.3) and on selection weekends (2.2). The conclusion was that further criteria not having been collected are more decisive for the selection decision.

The present studies aimed at identifying implicit selection criteria applied by assessors during the different stages of the selection procedure (when evaluating the statements of purpose, during the selection weekend and during the concluding discussions of assessors), as well as at answering the question whether an internal consistency of these implicit criteria may be verified for the different stages.

## Methods

At the time the studies were conducted, the UW/H selection procedure consisted of two stages: From approximately 1,000 applications per term in the form of a statement of purpose (1^st^ stage) 144 applicants with the highest score were invited to one of three selection weekends (2^nd^ stage), during which they participated in individual interviews and group discussions. During the assessors’ concluding discussions at the end of each selection weekend a ranking list was compiled according to the scores of the applicants. Subsequently, the first 42 applicants on the list were admitted to the medicine model curriculum at UW/H.

### Statements of Purpose in Extreme Group Comparison 

In order to present the implicit assessment criteria when evaluating the statements of purpose (1^st^ selection procedure stage) the statements of purpose of 20 admissions and rejections each were randomly selected for extreme group comparison from the applications for the 2010/11 winter term. Rejections were awarded a maximum of one point by assessors and therefore did not receive an invitation to the selection weekend. After admissions had been invited to the weekend due to a high score (9-10 points) they were awarded by assessors with at least 14 of 15 points on the selection weekend. The applicants’ statements of purpose were qualitatively tagged with codes and analyzed on the basis of grounded theory methodology [26], [27]. Saturation of categories was reached at 12 admissions and 18 rejections. As only one person [28] had tagged the data with codes and analyzed it, three additional independent persons tested the coding guidelines using one curriculum vitae as an example. This test confirmed the method and a comparable coding by a good reliability coefficient (Krippendorff’s α=0.857) [29]. The qualitative results of the content analysis were quantified as weighting and frequency of mentions, as well as in case of the “reflections” aspect by the extent of the same, i.e. the number of written lines. The latter aspect only became noticeably apparent during document analysis and was therefore used additionally. Subsequently, statistical evaluations (Mann-Whitney U tests) were performed for quantitative comparison of the two groups (admissions versus rejections).

#### Expert Interviews on the Selection Weekend

**Figure 1:**
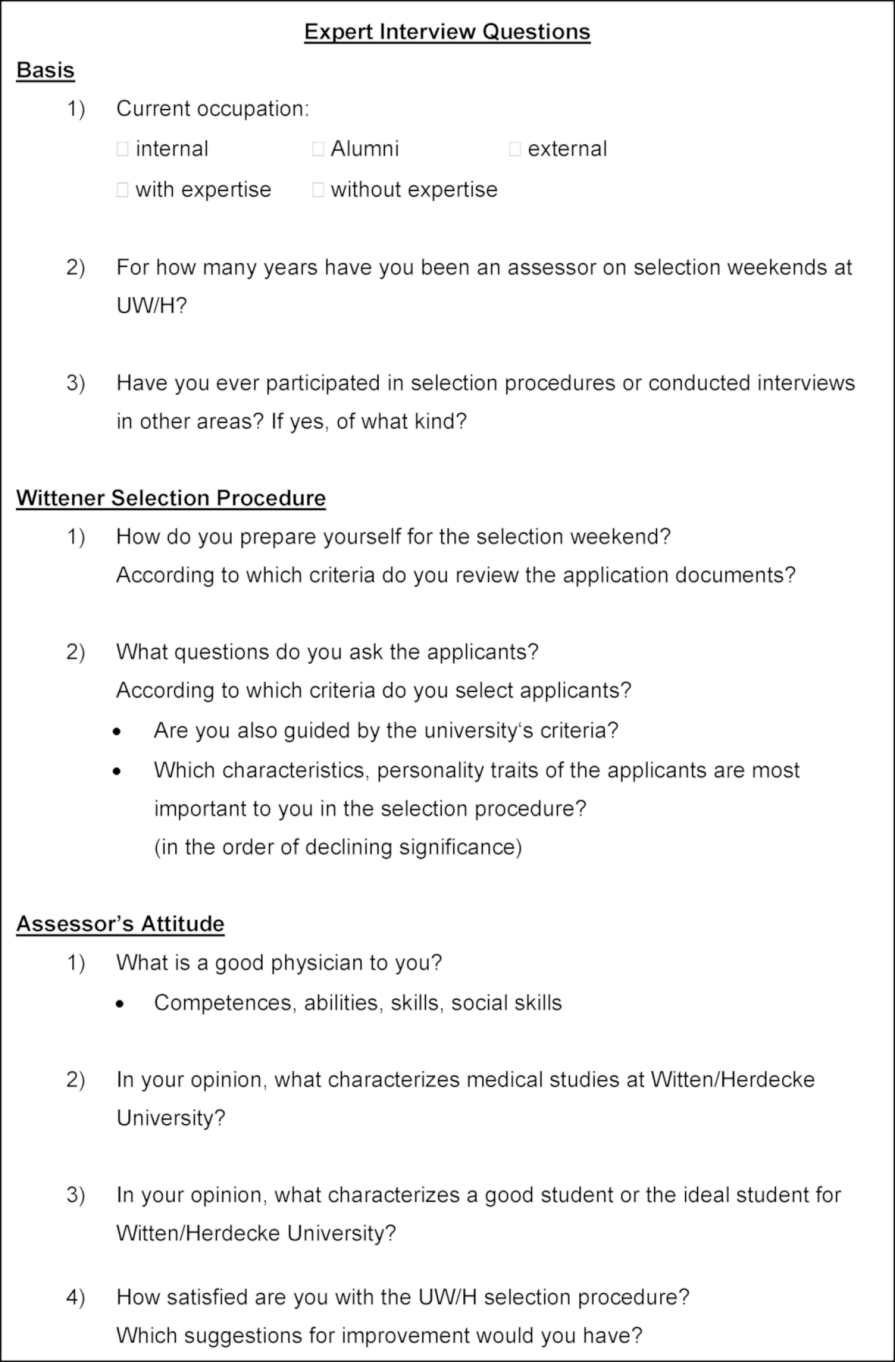
Guideline for Expert Interviews [32]

The Faculty’s cover letter addressed to assessors explicitly states the following assessment criteria: intellectual ability, reflection on study contents and conditions, social competence, commitment and extra-curricular activities. For gathering the implicit assessment criteria applied by assessors during the selection weekends as well (2^nd^ stage of the selection procedure) semi-structured, personal expert interviews were conducted [30], [31] until saturation of categories was reached with 25 of a total of 69 assessors in the 2010 summer term. Interviews with assessors were conducted by means of a guideline (see figure 1 (Fig. 1)) and did not take place during the selection weekends. 

In addition to stating demographic data assessors were expected to portray their approach to preparing and conducting the selection interviews, describe their implicit assessment criteria and name personality traits of applicants they consider important. The interviews, the duration of which varied significantly and which were an average of 45±22 minutes long, were transcribed, tagged with codes and categorized. The methodology was verified in this case, too, and a good intercoder reliability of Krippendorff’s α>0.800 was determined [32].

#### Focus Group Analysis of Concluding Discussions

By means of focus group analyses of two assessor groups with eight participants each the study examined whether there were areas of congruence between both groups despite subjective interpretation of the specified assessment criteria in the concluding discussions on the different selection weekends (2^nd^ stage of the selection procedure) in the 2011 summer term. Both group discussions lasted approximately 70 minutes, and were recorded and transcribed, followed by content analysis [33]. In addition to descriptive statistics, the agreement regarding the scoring system in both assessor groups were compared using the Mann-Whitney U test.

The Ethics Committee of Witten/Herdecke University has voted in favor of using extreme group comparison for the statements of purpose (88/2010). On request, the Ethics Committee saw no need to vote with respect to conducting expert interviews and focus group analyses. 

## Results

### Statements of Purpose in Extreme Group Comparison 

The final coding guideline for the statements of purpose with saturation reached at N=12 admissions and N=18 rejections consisted of 14 categories which were significantly pre-structured by an application’s formal background:

1. biographical data,
2. school career,
3. reasons for becoming a physician,
4. point of time at which this aspiration emerged,
5. social commitment,
6. activities/education and training before starting a degree course,
7. applications for medical studies,
8. interests,
9. reasons for applying at UW/H,
10. traits/skills,
11. objectives,
12. role models,
13. reflections,
14. concluding sentences.

The following presentation of results is limited exclusively to the categories showing significant differences between admissions ***[Admissions]*** and rejections ***[Rejections]***.

#### Category 2) School Career

In this category, interaction/commitment and personal initiative during schooldays, school internships, higher education entrance qualification grade and choice of subjects were tagged with codes (32 mentions; average sum 1.45±0.97). School career description and reflections thereon considerably deviate between admissions and rejections. In case of admissions, 58.3% (N=7) do not mention their school career at all, and the remaining 41.7% mention it only briefly (N=3) or in detail (N=2). In contrast, 58.3% (N=7) of rejections mention their school career in detail; the remaining 41.7 % mention it only briefly (N=9) or not at all (N=2). The rejections’ more extensive descriptions without concomitant reflection were rated significantly less favorable by assessors (see table 1 (Tab. 1)).

The higher education entrance qualification grade, which was rated by assessors as a sign of scholastic aptitude, showed a significant difference between the two groups (see table 1 (Tab. 1)). Seven admissions (23.3%) had a 2.0 higher education entrance qualification grade point average or better. No difference was found in the range of grades from 2.0 to 2.9. From a grade of 3.0, all applicants were rejected (N=3; 10%). 

##### Category 4) Point of time at which the aspiration to become a physician emerged

In this category, the emergence of the career aspiration was tagged with a time code, i.e. during elementary school/adolescence/sixth grade, community service/voluntary social year/internships, vocational training or through medical consultation or illness (28 mentions; average sum 1.39±1.13). Aspirations emerged during vocational training in case of the following anchor example of a rejection:

*“For already during the training as a nurse I felt the urge to be able to help my patients more comprehensively and to be allowed to assume more responsibility (…).” ****[Rejection 12]***

as well as in case of this admission where aspiration was caused by a case of illness in the family: 

*“A serious event was the reason for choosing a profession enabling me to socially interact with people, save lives, relieve pain, cure illnesses and make ailments more bearable.” ****[Admission 11]***

The emergence of the career aspiration caused by medical consultations during an illness was exclusively tagged with codes in case of admissions (N=2, 6.7%) and can therefore only be rated qualitatively. However, it is not the point of time or the origin of the career aspiration that is essential to assessors but rather subsequent reflection on and analysis of this aspiration.

##### Category 9) Reasons for Application

This category pools the reasons for applying specifically at UW/H (model curriculum; development of a discriminating medical personality capable of learning; additional education in complementary medicine or problem-oriented learning) (30 mentions; average sum 3.13±2.66) [24]. The development of a medical personality (N=3, 10%) and complementary medicine (N=4; 13.3%) were tagged with codes only in case of admissions. Qualitative differences in reflection became apparent with respect to problem-oriented learning and Studium fundamentale as reasons for applying at UW/H. This is reflected by the anchor example of an admission regarding practice-oriented teaching:

*“I regard the possibility to practically experience examinations already during my studies as a unique opportunity to prepare myself for interaction with patients before the clinical stage.” ****[Admission 6]***

as well as by the example of a rejection regarding the model curriculum:

*“Practice-oriented learning as well as learning in smaller groups, the examination formats of the model curriculum (…) are much more effective, varied and attractive for each prospective physician.” ****[Rejection 9]***

A significant difference between admissions (44 mentions) and rejections (36 mentions) can be verified regarding the extent of reflection as admissions describe their reasons for applying at UW/H more often and more extensively (Mann-Whitney U test: U= 46; p=.007). This is rated more favorable by assessors. 

##### Category 13) Reflections

In this category, the statements on a subject area relevant to the application were tagged with codes (32 mentions; average sum 1.33±1.21). The intensity of reflection was operationalized via the length of reflection, i.e. the number of lines, facilitating a quantitative analysis in addition to content analysis. This quantifiable aspect only became apparent during document analysis and was thereupon taken into account. Reflections are detectable in case of admissions (average sum 20.67±15.03) and in case of rejections (average sum 4.44±6.56). Reflections on the career aspiration (Mann-Whitney U test: U=49; p=.008) and the length of the statement of purpose (Mann-Whitney U test: U=13; p=.000) showed significant differences between the two extreme groups, which were relevant with respect to content analysis and statistically significant:

*“Shortly before taking the school leaving examination I began to have my doubts whether I was able to cope with the responsibility of a medical profession. (…) But my family and my friends tried to take away this fear and, in the end, I regained my confidence and would now like to study medicine more than ever before in order to be able to help people in the future.” ****[Admission 3]***

In case of admissions the length of the statement varied from 1.5 to 4 pages with an average of 2.79±0.89 pages, while in case of rejections more than 60% (N=11) applied with a statement of purpose of one page maximum (1.16±0.63 pages; range 0.5 to 3 pages). 

#### Expert Interviews on the Selection Weekend 

The semi-structured survey was conducted according to the interview guideline (see figure 1 (Fig. 1)) and included 25 assessors (18 male, 7 female), 17 of which were physicians. The average age was 44.6±9.5 years. The percentage distribution of men and women represents the whole group of assessors participating in the UW/H selection procedure with a percentage of women of approximately 30% [32]. About half of the assessors (N=14) were university employees; 11 assessors worked in associated hospitals. Assessors have participated in the UW/H student selection procedure for two to 22 years.

The content analysis identified three main categories in the expert interviews: intellectual ability, motivation and social competence. Each main category consisted of a minimum of two subcategories as shown in table 2 (Tab. 2). The categories contain various aspects which were either determined during the interview (e.g., motivation for becoming a physician) or had to be interpreted based on observable behavior and applicants’ presentations (e.g., non-verbal communication, maturity, identity formation). 

Intellectual ability was the category most mentioned by assessors (215 mentions; average sum 7.24±4.87), with ability being understood in this context as solidified system of generalized mental processes that control the carrying out of activities and thus enable performance [34]. This category combines twice as much subcategories as the other two main categories (applicants’ examination performance, in particular higher education entrance qualification grade; communication skills; (self-) reflection/maturity and logical reasoning skills). The anchor example of the maturity subcategory reflects the thoughts of the assessor: 

*“And where we have to decide whether the persons applying here are probably/presumably able to cope with it.” ****[Assessor 8]***

The motivation category was the second most mentioned one (average sum 3.44±1.78; 190 mentions in total). A distinction was made between motivation for the profession, with N=113 by far the most frequently mentioned subcategory, and motivation for medical studies at UW/H. The following anchor example underlines the latter aspect mentioned by assessors: 

*“Well, I had already mentioned before that of course I ask for reflection on Witten/Herdecke University, including the mission statement on truth, freedom, social responsibility.” ****[Assessor 16]***

In the main category of social competence 123 mentions by assessors were tagged with codes (average sum 1.40±0.89). This category combined the subcategories interpersonal skills/group skills reflecting primary competencies [35] such as the ability to adopt the perspective of others, self-control and assertiveness, as well as extra-curricular activities as an expression of value pluralism, self-presentation and competition. The differentiating aspect of social competence in the meaning of permanent social commitment based on the primary competencies prosociality and supporting others becomes apparent in the following anchor example:

*“And regarding social commitment I do ask them what they do in their spare time, whether they are involved in any clubs, associations, parties or whatsoever or in local communities in one way or another. I let them describe in detail what exactly they are doing there in order to find out whether they are really active there or whether they just show up at the annual general meeting once a year.” ****[Assessor 4] ***

The weighting of the categories indicates how extensively assessors talked about each category. If assessors outlined aspects of the category across several sentences, each new aspect of the subject matter was rated regardless of whether it belonged to the same sentence or another. Again, motivation for the medical profession was the most important subcategory (441 mentions), followed by (self-) reflection (272 mentions), motivation for studying at UW/H (216 mentions) and extra-curricular activities (215 mentions). The following anchor example regarding motivation for the medical profession shows that the ability to reflect as general content category is crucial for assessors and that it resonates in other categories as an applicant’s basic competence:

*“(….) to say, especially if the candidate’s CV implies it: ‘You have just completed your nursing education, what’s the difference to working as a doctor?’” ****[Assessor 7]***

#### Focus Group Analysis of Concluding Discussions

Focus group analyses were used to examine the concluding discussions on two selection weekends (2^nd^ stage of the selection procedure). Five assessors of focus group 1 mainly work in patient care, and three assessors are primarily active in teaching. Focus group 2 consisted of four assessors working in patient care and four assessors working as teachers. With six males to two females the gender ratio is the same in both groups. 

The content analysis identified four main categories based on the discussions in both focus groups: performance, personality, personal growth potential and ability to reflect. Each main category consisted of two to four subcategories, as indicated in table 3 (Tab. 3). The subcategories cover different personality traits and characteristics of the applicants, stated by assessors as evaluation basis. 

In the performance main category 59 mentions of assessors were tagged with codes (average sum 3.62±3.32), while differentiating between performance during the presentation of a topic of their own choice, performance regarding behavior in debate/group contribution and the formal curriculum vitae. The following anchor example illustrates the positive assessment of a group contribution by an assessor: 

*“(…) has also moderated the debate very well. There was much teamwork, much debate and she has nicely picked up lines fed to her and passed them on as well.” ****[Assessor B]***

Focus group assessors most frequently mentioned the personality category (average sum 5.50±4.45; 87 mentions in total), which comprises four subcategories and thus the most ones: charisma, sincerity, mental flexibility and emotional stability. A negative mark in the sincerity category reflects an applicant’s disharmonious expression as the following anchor example shows:

*“(…) I had the impression that he had sprinkled his CV with it again and again, maybe because he wanted to play to the gallery or because he believes to be able to get on this way. (…).” ****[Assessor N]***

In the main category of personal growth potential the least mentions of assessors were tagged with codes (average sum 2.56±2.89; 41 mentions in total). This category combines motivation, potential and social competence as subcategories. The following anchor example illustrates an attitude rated as favorable with respect to motivation:

*“He will go through it, he will learn, he wants to expand his horizon.” ****[Assessor P]***

Only two subcategories were assigned to the ability to reflect as a main category: reflection on the profession and immaturity or naivety. They account for 70 mentions in total at an average sum of 4.44±3.56. The following anchor example is an example of a statement rated as reflection:

*“But what she said was really well-founded. I think it contained an incredible number of interfaces to other topics; it was phrased very openly, and she herself had quite some inner space there, I found.” [****Assessor R]***

**Figure 2:**
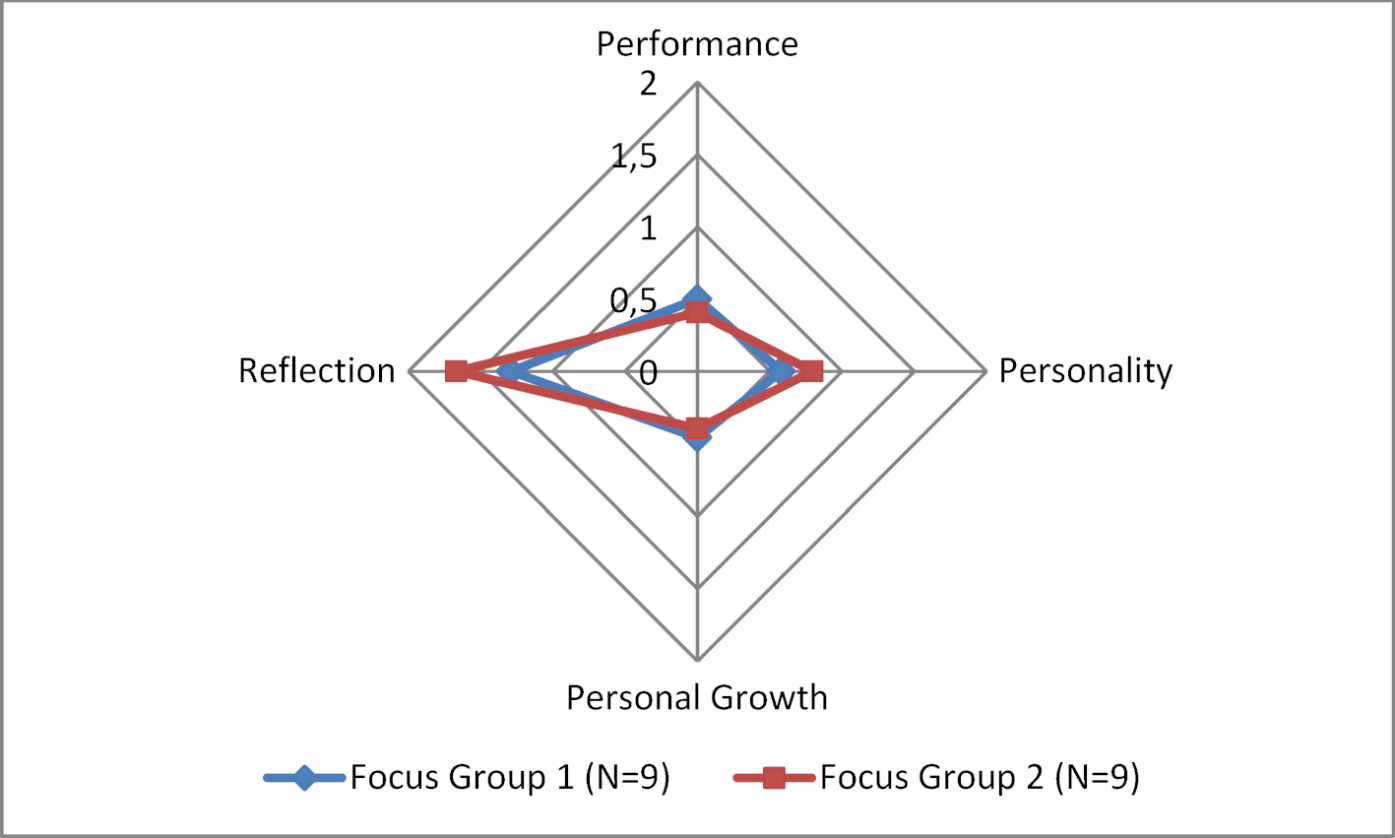
Frequency of Categories in the Focus Groups (N=9 each) [33]

In summary, most mentions were made in the main category of personality, owing to the fact, however, that this main category has the most subcategories. After having corrected the unequal distribution by dividing mentions in the four main categories by the number of related subcategories (cf. table 3 (Tab. 3)), the following frequencies result as shown in figure 2 (Fig. 2). In order to enhance comparability, the frequency of mentions in the main categories was transformed. 

The ability to reflect was thus mentioned most frequently as an assessment criterion in both focus groups on the two selection weekends with different assessors. The other three main categories show a similar distribution within the focus groups.

## Discussion and Conclusions

The results of the qualitative studies on the selection procedure for medical studies at UW/H reflect the framework conditions of the respective procedure stage. The categories extracted by content analysis from the extreme group comparison between admissions and rejections regarding statements of purpose are significantly pre-structured due to their formal background [28]. The expert interviews on the implicit assessment criteria of assessors on the selection weekends revealed that they were guided by the explicit UW/H criteria and showed, respectively, which contents were attributed to these criteria [32]. In addition, the different weighting of the assessment criteria by assessors was demonstrated again by the focus group analyses of the two concluding discussions [33]. The present studies conducted in the context of assuring the selection procedure’s quality clearly indicate the following main assessment categories: motivation for the profession and for studying at UW/H; performance and scholastic aptitude; personality, personal growth potential and social competence, as well as the ability to reflect as basic competence and general category. 

### Motivation

The extreme group comparison showed that admissions mentioned the origin of career aspirations (medical consultations; illness; role models) and reasons for applying at UW/H (development of a medical personality; additional training in complementary medicine; problem-oriented learning) more often than rejections [28]. During the expert interviews the subcategory of motivation for the medical profession was mentioned by far most frequently by assessors [32]. They also confirmed that the applicants’ motivation for the medical profession [36] and studying at a specific institution [11] belonged to the key and mandatory questions of a selection interview. The use of this selection criterion as valid predictor of future academic performance could be confirmed by a review of 56 studies [37]. In addition to the impact of motivation on students’ learning, motivation in terms of the self-determination theory of Deci and Ryan [38] is also a dependent variable which may be influenced by autonomy, competence and relatedness. Correspondingly, the motivation selection criterion is thus to be found as a subcategory of the personal growth potential in the present focus group analyses [33]. The Master Plan Medical Studies 2020 [39] suggests systematically taking greater account of motivation for medical studies as a selection criterion in future selection procedures. A longitudinal analysis of the development of students’ motivation in the course of studies considering the theoretical assumptions of the self-determination theory seems to be predestined for future studies.

#### Performance

The present extreme group comparison substantiated a significant difference between the two groups with a considerably better higher education entrance qualification grade point average in case of admissions [28]. The higher education entrance qualification grade as a predictor of intellectual ability was important to 15 assessors in the expert interviews, but poorer grades were no criterion for rejection for 10 assessors as long as applicants were able to give an explanation for their poor academic performance on request during the biographical interview [40] and provided the scholastic aptitude was taken for granted [32]. However, it was not the narrative being important to assessors, but rather the applicants’ reports on their change of behavior and accompanying reflection on their professional career after having achieved a poor higher education entrance qualification grade. Applicants who just reported repeatedly and more elaborately on their school career with all its chances and difficulties were admitted less often [28]. 

In the concluding discussions [33] the applicants’ performance regarding the formal curriculum vitae, group discussions and presentation was also assessed in a results-oriented approach. On the national and the international scale it is recognized that the higher education entrance qualification certificate is a positive predictor of successful (preclinical) studies [14], [41]. It proves the applicant’s ability to deliver the required cognitive performance in the context of public examinations. The correlation between school leaving grades and study performance is even stronger the more school-like university teaching is [42], which applies to the preclinical section of medical studies at many universities [14], [15]. 

#### Personality

The extreme group comparison revealed that the educational objective of UW/H, i.e. the medical professional’s lifelong capability of learning, was mentioned as a reason for application more often in case of admissions. Applicants who reflected significantly more intensely on their commitment, previous education or courses of studies were admitted more frequently [28]. Extra-curricular activities were mentioned as important criterion by almost all assessors during the expert interviews [32]. This was expressed particularly in connection with the aspiration to select “outstanding personalities” being committed beyond the normal level. Moreover, this subject area of social commitment was used to check the applicants’ ability to self-reflect. Assessors who participated in the present focus group analyses describe the personality criterion using charisma, sincerity, mental flexibility and emotional stability as subcategories. Further personality traits are to be found in the personal growth potential category, such as social competence [33]. Nevertheless, a systematic review on the methods used in selection procedures established the fact that there is no evidence of a correlation between personality traits and medical performance [43]. While the standardized personality trait of diligence was mentioned in some studies as a positive predictor of examination performance, other studies view it as a negative predictor of some aspects of clinical performance. Consequently, the association between personality traits and performance in medical education and the subsequent medical career is rather complex and possibly non-linear. Personality traits should therefore not be a primary criterion in selection procedures for the time being and rather be perceived as additional assessment criterion. 

#### Reflection

The research question of the present extreme group comparison can be clearly answered after content analysis of the applicants’ statements of purpose: The ability to reflect is the key difference between admissions and rejections [28]. In the expert interviews, too, the applicants’ maturity and ability to reflect were mentioned by UW/H assessors as a separate competence as well as a competence overlapping with all other categories [32]. Both focus group analyses produced the same result with the ability to reflect as the most influential selection criterion [33]. This result confirms the internal consistency of this implicit criterion over the different stages of the selection procedure for medical studies at UW/H. The ability to reflect could therefore be clearly isolated as an aspect leading to an increased probability of successfully passing both stages of the selection procedure. 

After all, the ability to reflect is one of the key competencies required for medical education and professional medical action [44], [45]. A systematic review evaluates reflection and the ability to reflect in the medical context [46]. On the one hand, medical students are expected to acquire this ability in the course of their studies and, on the other hand, curricula attempt to promote the development of reflective thinking. UW/H, too, pursues this dual approach by further developing and exercising the reflective applicants’ ability to reflect in the context of the accompanying Studium fundamentale [23] and mentoring program [47].

#### Limitations 

The results presented by this paper are based on the results of three qualitative studies with a comparatively low number of cases and yet a certain degree of heterogeneity in the applicant and assessor groups. Moreover, only the best and the worst statements of purpose were used for extreme group comparison (only approx. 2% of an application cycle in total), constituting a loss of information and thus limiting the generalizability of the results. 

## Conclusions

The studies conducted for assuring the quality of the selection procedure for medical studies have yielded a clear result with regard to the selection criteria and in the light of the UW/H profile and objectives. So far, the UW/H selection procedure has appeared to be comparable to the procedures of other medical faculties to a limited extent only. However, the present results show that the criteria identified are congruent with national as well as international literature and also anticipate aspects of the Master Plan Medical Studies 2020, such as the recommended identification of social and communication skills and an extraordinary motivation for medical studies [39]. The recent decision of the German Federal Constitutional Court on the allocation of places to study medicine also demonstrates that the higher education entrance qualification grade cannot continue to be the only criterion for ensuring equal opportunities [http://www.bverfg.de/e/ls20171219_1bvl000314.html]. This is why this study on implicit criteria for selecting suitable applicants for medical studies has become even more topical. 

The ability to self-reflect requires reflection on and communication of professional challenges and can be deemed as principal predictor of lifelong professional development, research-based learning and successfully practicing medicine [47]. The aim of the UW/H selection procedure is to educate good physicians who acquire a broad, scientifically sound knowledge and social skills, a high proportion of whom work in the field of patient care [23] and who, above all, are able to self-reflect. 

## Acknowledgements

The authors like to thank Michaela Munk for the professional translation.

## Competing interests

The authors declare that they have no competing interests. 

## Figures and Tables

**Table 1:**
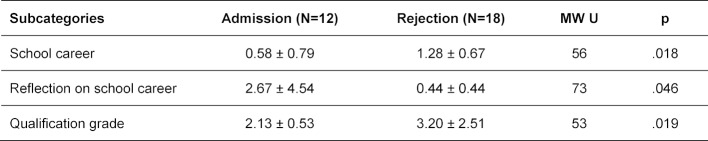
Mentions regarding school career, reflection on school career and higher education entrance qualification grade (sum, mean value, standard deviation, Mann-Whitney U test, p) [28]

**Table 2:**
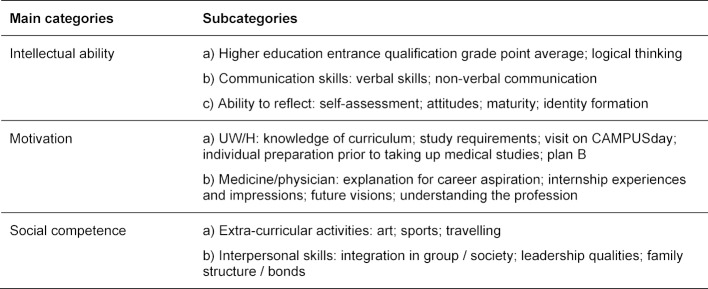
Main categories and subcategories extracted from expert interviews by means of content analysis [32]

**Table 3:**
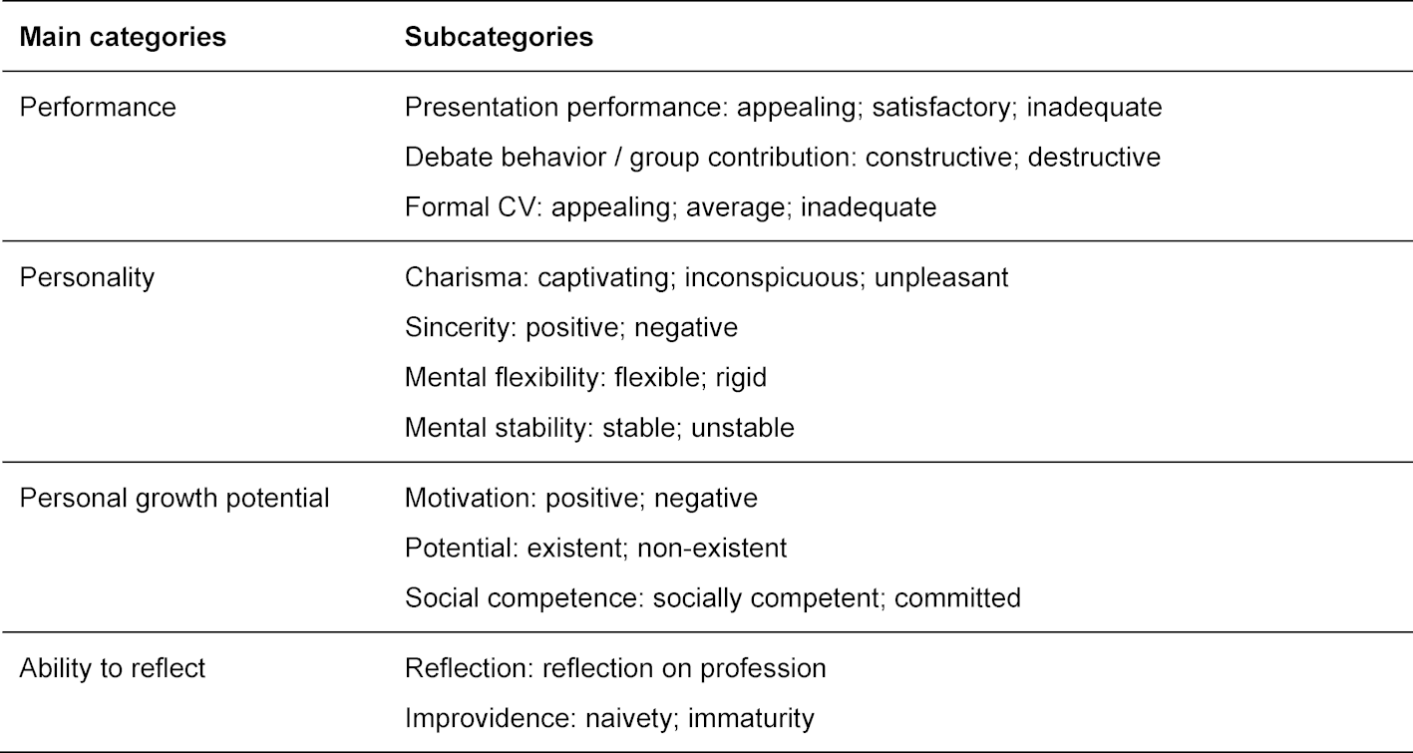
Main categories and subcategories extracted from focus group discussions by means of content analysis [33]
